# Fish Intake and Risk of Liver Cancer: A Meta-Analysis

**DOI:** 10.1371/journal.pone.0096102

**Published:** 2015-01-23

**Authors:** Rui-Xue Huang, Yan-Ying Duan, Jian-An Hu

**Affiliations:** Department of Occupational and Environmental Health, School of Public Health, Central South University, Changsha, Hunan Province, China; Van Andel Institute, UNITED STATES

## Abstract

**Background:**

Increasing laboratory findings indicate that n-3 fatty acids, mainly derived from fish, inhibit cancer development and progression, but results from epidemiologic studies have been inconsistent and inconclusive.

**Objective:**

To evaluate the association of fish intake with risk of liver cancer by conducting a meta-analysis.

**Methods:**

Published case-control/cohort studies that evaluated the relationship between total fish intake and risk of liver cancer were found on PubMed and EMBASE. The pooled relative risks (RRs) with 95% confidence intervals (CIs) were obtained with the random-effects model.

**Results:**

Five retrospective case-control studies and 5 prospective cohort studies were included in the final analysis, involving a total of 3 624 liver cancer cases. Comparing the highest with the lowest category of total fish intake, the pooled RRs of liver cancer were 0.79 (95% CI, 0.59-1.06) for case-control studies, 0.82 (95% CI, 0.70-0.96) for cohort studies and 0.82 (95% CI, 0.71-0.94) for all studies combined. The protective effects of total fish intake against liver cancer were confirmed by stratified and sensitivity analyses. In addition, an increase in fish intake of 1 serving/week was estimated to be significantly associated with 6% lower risk of liver cancer (RR = 0.94, 95% CI, 0.91-0.98).

**Conclusions:**

Findings from this meta-analysis suggest that a higher fish intake is associated with reduced risk of liver cancer.

## Introduction

Liver cancer is the sixth most frequently diagnosed cancer and the third most common cause of cancer-related death worldwide, with about 564 000 new cases occurred each year [1,2]. The burden of this disease is likely to continue to increase until 2030 [1,3]. In spite of a number of well-established risk factors for liver cancer, including chronic infection with hepatitis B virus (HBV) or hepatitis C (HCV), less attention, however, has been paid to the role of dietary factors except for alcohol abuse and aflatoxin contamination in the development of this malignancy [2,4,5,6].

Fish is a rich source of n-3 polyunsaturated fatty acid (n-3 PUFA). In a previous review addressing potential mechanisms where by n-3 PUFAs may affect cancer risk, Larsson *et al*. pointed out that “mounting evidence shows that dietary n-3 PUFAs inhibit the promotion and progression stages of carcinogenesis”[7]. Conversely, epidemiologic findings regarding the relationships between fish intake and cancer risk are inconsistent and inconclusive [8]. The 2007 report from the World Cancer research Fund and American Institute for Cancer research concluded that the evidence supporting the benefits of fish on cancer risk was “limited to suggestive” and based mainly on studies of colorectal cancer [8]. However, a large body of new evidence has emerged since the report. In particular, a number of case-control [9,10,11,12,13] and cohort [14,15,16,17,18] studies that investigated the role of fish intake in the development of liver cancer have been carried out, but the results remain inconsistent. The purpose of this study was to investigate the association of total fish intake with risk of primary liver cancer by quantitatively summarizing the published case-control and cohort studies.

## Materials and Methods

### Literature search

We searched for potentially relevant publications through December, 2013 on PubMed and EMBASE databases using the search terms “fish” in combination with “liver cancer” or “hepatocellular carcinoma” or “liver neoplasm”, without language restrictions. The reference lists of retrieved publications were also carefully reviewed for any further studies.

### Study selection

Studies were eligible for inclusion if they met the following inclusion criteria: (1) study design was case-control or cohort; (2) exposure of interest was total fish intake; (3) outcome was incidence or mortality of liver cancer; and (4) relative risks (RRs) or odds ratios (ORs) with corresponding 95% confidence intervals (CIs) were reported (or could be estimated). Studies that investigated the effect of specific type of fish or fish prepared/cooked with specific methods (i.e., raw fish, salted fish or broiled fish) were excluded. The titles and abstracts of all potentially relevant publications were reviewed to evaluate the relevance of the information; full-texts were scrutinized if any potentially relevant information was identified in a retrieved abstract.

### Data extraction

The following data were extracted from each included eligible study using a standardized data-collection protocol: the first author’s last name, publication year, country of origin, study design, number of cases and subjects, outcomes (incidence or mortality), ascertainment of exposure and outcome, levels of fish intake, RR or OR estimates with corresponding 95% CIs for each category of fish intake and variables controlled for in the analysis. Literature search, study selection and data abstraction were performed independently by two investigators, with any disagreements resolved by discussions.

### Statistical analysis

RR with 95% CI is the measure of effect of interest in this meta-analysis, and ORs in the included case-control studies were considered as RR approximations because the risk of liver cancer is sufficiently low. Where possible, the risk estimates with adjustment for multivariables were used. The study by La Vecchia *et al*.[11] did not report 95% CIs for each category of fish intake, and the data were estimated according to the number of cases and non-cases provided. For the study by Kurozawa *et al*.[17] that reported RR estimates by history of liver diseases, age and gender, the data were combined with the fixed-effects model and the pooled results were included. The random-effects model taking into account both within- and between-study variation was assigned to compute the summary risk estimates [19]. Stratified analyses were carried out for the subgroups study design, geographic area, number of cases and outcome (incidence or mortality).

A dose-response analysis was performed according to the method proposed by Greenland and Longnecker [20] and Orsini *et al*.[21]. Because the included studies used different units to report fish intake (i.e., grams or servings), we rescaled intake into servings per week using 100 grams as the approximate average serving size[22]. For every study, the median or mean level of fish for each category was assigned to each corresponding risk estimate. If the median or mean level was not reported, we assigned to each class the dose corresponding to the midpoint of upper and lower boundaries. If the highest or lowest category was open-ended, we assumed the width of the interval to be the same as in the closest category.

Statistical heterogeneity was assessed using Q and *I* ^2^ statistics[23]. For the Q statistic, a *P-*value of less than. 1 was considered statistically significant heterogeneity. Potential publication bias was evaluated using Egger’s test and Begg’s funnel plot[24]. All statistical analyses were performed using STATA software, version 12.0.

## Results

### Literature search and study characteristics

**Figure 1:**
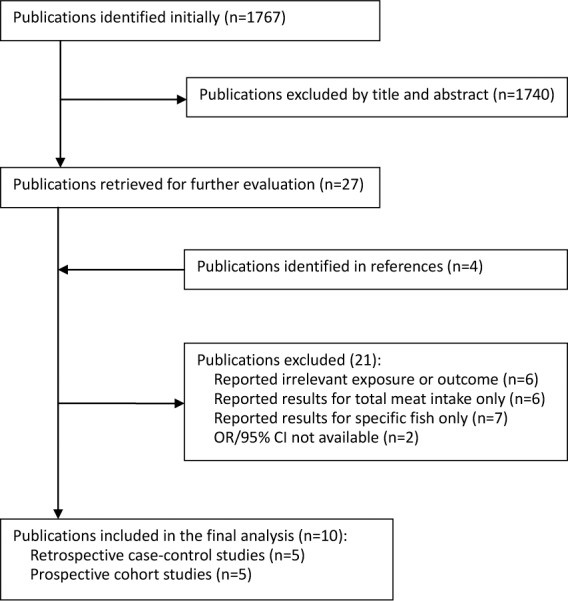
Flow chart of literature search.

The flow chart of literature search is shown in Fig. 1. Seven studies [25,26,27,28,29,30,31] examining the relationship between intake of specific type of fish and risk of liver cancer were rejected, and the characteristics of these studies were reported in S1 Table. Finally, 10 studies [9,10,11,12,13,14,15,16,17,18] that met the pre-specified inclusion criteria were included in this meta-analysis.

The 10 studies, including 5 retrospective case-control studies (all except for 1 study[13] were hospital based) and 5 prospective cohort studies, contained a total of 3 624 liver cancer cases. These studies were published between 1988 and 2013, of which three were conducted in Japan, 3 were performed in Italy, 1 was from the United States, 1 from Serbia, and the remaining 1 was a multicenter prospective study (the European Prospective Investigation into Cancer and Nutrition [EPIC] study) carried out in 10 European countries. The number of cases ranged from 45 to 1 116, and the number of subjects ranged from 135 to 492 186. The outcome of interest was liver cancer incidence in 6 studies, and was liver cancer mortality in 3 studies. The EPIC study reported combined results for cancer incidence and mortality. In the prospective cohort study by Hirayama *et al*.[16] that accessed the association of fish intake with liver cancer mortality, only the results from a sub-cohort consisting of patients with liver cirrhosis could be included in this meta-analysis. The methods for exposure assessment and cancer identification, and the variables controlled for among included studies were largely different. The study characteristics are summarized in S2 Table.

### High vs. low intake

**Figure 2:**
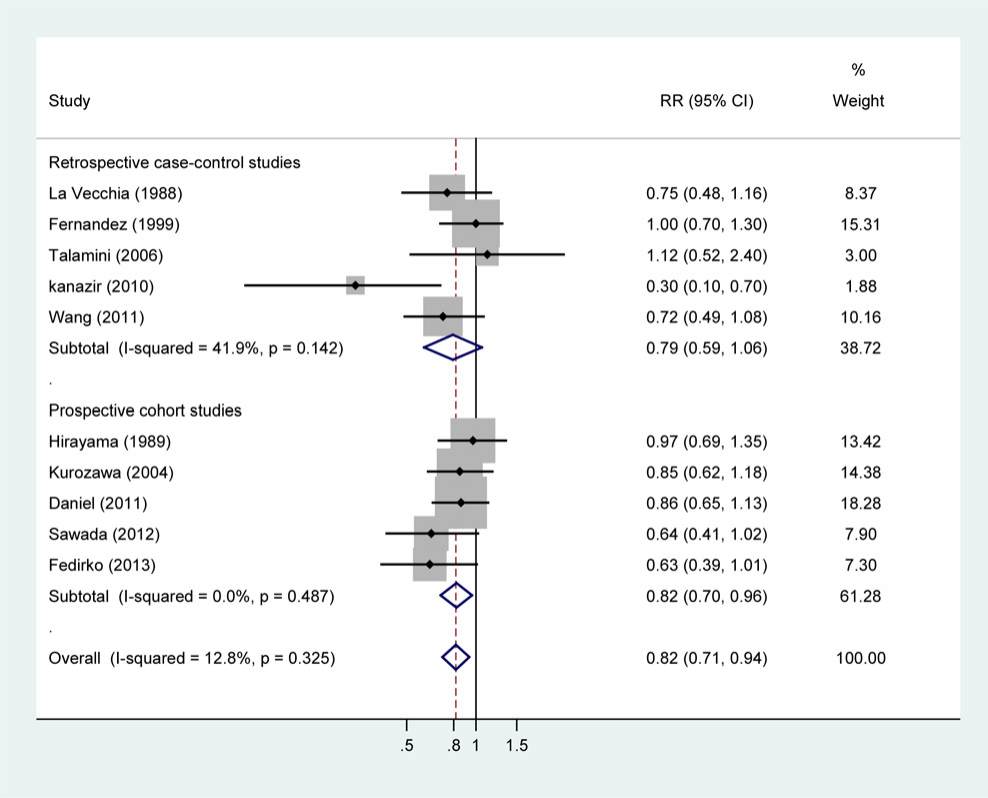
Relative risk (RR) of liver cancer for the highest compared with the lowest categories of fish intake. RRs of greater than 1 represent increased risk of liver cancer associated with higher fish intake, and RRs of less than 1 represent decreased risk.

The RRs for liver cancer with different fish intake categories relative to the lowest category are shown in Fig. 2. Compared with the lowest category of fish intake, the pooled RR for liver cancer was 0.82 (95% CI, 0.71–0.94), with little evidence of heterogeneity (*P* = 0.33, *I* ^2^ = 12.8%).

Stratifying the analysis by study design, the summary RR was 0.79 (95% CI, 0.59–1.06) for case-control studies and 0.82 (95% CI, 0.70–0.96) for cohort studies. According to geographic area, the summary RR was 0.81 (95% CI, 0.68–0.98) for 4 Asian studies and 0.77 (95% CI, 0.56–1.06) for 5 European studies. By the number of liver cancer cases, the pooled RR was 0.76 (95%CI, 0.56–1.04) for 5 studies with cases of ＜300 and 0.84 (0.72–0.97) for 5 studies with≥300 cases. The summary RR for liver cancer incidence was 0.81 (95% CI, 0.64–1.01) and for cancer mortality was 0.85 (0.70–1.04). A sensitivity analysis conducted by excluding one study at each turn and pooling results from the remainder showed that the summary RR ranged from 0.79 (95%CI, 0.69–0.91) to 0.84 (95%CI, 0.73–0.96), indicating that our findings were not sensitive to any individual studies.

The hospital based case-control study by Kanazir *et al*.[10] was an outlier, and excluding this study yielded a RR of 0.84 (95%CI, 0.74–0.95). Further omitting the sub-cohort by Hirayama *et al*. [16] that consisted of liver cirrhosis patients obtained a similar result (RR = 0.82, 95% CI, 0.72–0.94).

### Dose-response analysis

Seven studies [9,12,13,14,15,17,18] were included in the dose-response analysis. The summary RR of liver cancer for an increase in fish intake of 1 serving/week was 0.94 (0.91–0.98), with no heterogeneity (*P* = 0.74, *I* ^2^ = 0.0%). The inverse association remained statistically significant when restricting to prospective cohort studies (RR = 0.94, 95% CI, 0.90–0.98).

### Publication bias

**Figure 3:**
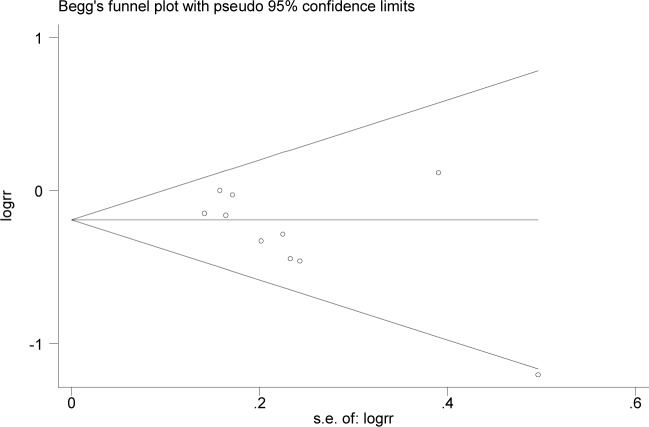
Begg’s funnel plot testing possible publication bias.

There was a suggestion of publication bias according to Egger’s test (*P* = 0.07). Visual inspection of the Begg’s funnel plot, however, did not show considerable asymmetry (Fig. 3). When the outlier[10] was omitted, *P* value for Egger’s test was 0.39.

## Discussion

In this meta-analysis of published case-control and cohort studies, we found a statistically significant inverse association between total fish intake and risk of liver cancer. Analyses of high vs. low intake and dose- response models indicated that liver cancer risk was reduced by 18% and 6% per 1 serving/week increase, respectively.

The exact mechanisms whereby fish intake reduces risk of liver cancer are not well-established. Nevertheless, fish is a rich source of n-3 PUFA, which may be of anti-carcinogenic and anti-inflammatory properties [7]. Experimental evidence suggests that n-3 PUFA may inhibit cancer development and progression by beneficially altering a wide range of mechanisms such as molecular biosynthesis, gene transcription and expression, and signal transduction [7]. Evidence from clinical trials found that dietary supplementation with n-3 PUFA reduced release of interleukin-1 and interleukin-6 [32]. Given the fact that liver cancer is an inflammation-related cancer, n-3 PUFA might protect against liver through its anti-inflammatory effect. The Japan Public Health Center-based (JPHC) prospective study [18] also showed significantly decreased risk of liver cancer in those with a higher intake of n-3 PUFA.

We were not able to clarify the effects of specific fish on liver cancer development because the findings were considerably heterogeneous (S1 Table). Presumably, differences in fish preservation and cooking styles may be an important determinant in the heterogeneity.

We had also attempted to investigate whether the association between fish intake and risk of liver cancer was confounded or varied by status of hepatitis viruses. However, few data were available addressing this issue. One hospital based case-control study [12] showed a neutral result after adjustment for hepatitis viruses (OR = 1.12, 95% CI, 0.52–2.4). In the JPHC study [18], the apparent protective effect of total fish intake upon liver cancer risk actually became stronger in magnitude after adjusting for HCV and HBV antigen (HBsAg) status, although the result was no longer statistically significant due to the concomitant loss of power (RR = 0.54, 95% CI, 0.23–1.24). In a case-control subset nested in the EPIC study[15], the potentially protective effect of total fish on liver cancer was not altered by adjustment for HBV/HCV. More large prospective studies taking into account hepatitis viruses status are required.

Possible biases from case-control studies, such as selection bias and recall bias are of concern. However, the rate of declining participation in cases and subjects were generally low (i.e.＜3% [9,10,12]), indicating that selection bias is unlikely to be a serious problem. There also was no reason to assume different recalls of fish intake on the basis of disease status, because fish was not commonly considered to protect cancer at the time of those case-control studies. In addition, the findings of cohort studies, which generally give a higher level of evidence, were very similar to those of case-control studies. Taken together, selection and recall biases are unlikely to have materially altered our findings.

A further important consideration is the potential publication bias. It is well known that positive results have a greater chance of being published, whereas small studies with null findings tend not to be reported. Two commonly applied tests to access publication bias are Begg’s and Egger’s tests. In an overall meta-analysis of case-control and cohort studies, there was a suggestion of publication bias with Egger’s test (though not significant). However, the test may be of low statistical power when the number of studies was relatively small[33]. Thus, we gave more emphasis on an informal visual inspection of the funnel plot. Small studies with null results would be expected to fall in the upper right quadrant of the plot, and there was indeed one study located in this area. Furthermore, after an outlier was excluded, there was no evidence of publication bias, and the pooled results did not change substantially (RR = 0.84, 95% CI, 0.74–0.95). These observations minimize the possible impact of publication bias on our findings.

Several limitations in this meta-analysis warrant mention. Residual or unknown confounding cannot completely be ruled out. Those subjects with higher fish intake may also be more likely to have favorable dietary patterns, healthier lifestyles, or both. Several included studies also did not control for a variety of potential risk factors for liver cancer, such as HBV/HCV, alcohol and coffee drinking, smoking and body mass index, etc. Second, the levels of fish intake among different populations, and the methods for assessing fish and identifying liver cases were largely inconsistent. However, there was little evidence of heterogeneity either in the high vs. low, or in the dose-response analyses. Finally, we only investigated total fish, and the effects of individual fish on liver cancer risk remains to be determined in future studies. The potential limitations for observational studies to access relationship between diet and health, and the difficulty in conducting prospective studies properly accounting for multiple risk factors for liver cancer were also well addressed in the editorial by Freedman and Marrero[34]. As they pointed, although fish intake currently cannot be recommended for liver cancer prevention, “these provocative results merit future study”.

In sum, findings from this meta-analysis of published case-control and cohort studies suggest a significant inverse association between total fish intake and risk of liver cancer. Future well-designed prospective studies are needed to further confirm our findings.

## Supporting Information

S1 PRISMA Checklist PRISMA 2009 checklist in this meta-analysis. (DOC)

S1 Table Characteristics of excluded case-control and cohort studies on specific fish intake and risk liver cancer. (DOCX)

S2 Table Characteristics of published case-control and cohort studies on total fish intake and Risk of liver cancer. (DOCX)
