# Supplementary material for: Fish Intake and Risk of Liver Cancer: A Meta-Analysis
Source: PLoS One. 2015 Jan 23;10(1):e0096102. doi: 10.1371/journal.pone.0096102 (PMC4304705; doi:10.1371/journal.pone.0096102)
Supplement: S1 Table — (DOCX) [file pone.0096102.s002.docx]

| Study | Country | Design | Cases/  subjects | Outcomes | Comparison | Type of fish | OR /RR (95% CI) | Variables controlled for |
| --- | --- | --- | --- | --- | --- | --- | --- | --- |
| Lam, 1982 | China | Hospital based case-control | 107/214 | HCC incidence | ≥1 vs.＜1 times /wk | Salted fish | OR = 1.1 | Age and sex. |
| Ikeda, 1983 | Japan | Prospective cohort | 24/7553 | Liver cancer mortality | ≥2 vs.＜2 times /wk | Dried fish  Broiled fish | RR = 2.75  RR = 1.32 | Age, sex, radiation dose, smoking, school career, and consumption of rice, fruit, salted pickle and milk. |
| Lu, 1988 | China | Hospital based case-control | 131/338 | HCC incidence | Often vs.  Occasional/never | Raw fish | OR = 1.19 | Age, sex and HBsAg |
| Strivatanakul, 1991 | Thailand | Hospital based case-control | 65/130 | HCC incidence | ≥3 vs.＜3 times/d | Pla-chom (fermented fish) | 0.5 (0.2-1.0) | Age, sex and residence. |
| Wang, 1996  (in Chinese) | China | Hospital based case-control | 96/240 | liver cancer incidence | Ever vs. never | Raw fish  Salted fish | 3.23 (1.70-6.13)  2.34 (1.12-4.91) | Age, sex, residence, alcohol drinking, HBsAg, and history of liver disease |
| Yu, 2002 | China | Population  based case-control | 248/596 | HCC incidence | ≥2 vs.＜2 times/mo  ≥3 vs.＜3 times/mo | Salted fish  Fresh fish | 0.61 (0.26-1.43)  0.31 (0.13-0.71) | Age, sex, residence and HBV. |
| Qiu, 2008  (in Chinese) | China | Population based case-control | 500/1000 | liver cancer incidence | Ever vs. never | Raw fish | 7.71(5.00-12.0) | Age and sex. |

**Table S1** . Characteristics of excluded case-control and cohort studies on specific fish intake and risk liver cancer.

CI, confidence interval; HBsAg, hepatitis B virus antigen; HBV, hepatitis B virus; HCC, hepatic carcinoma; OR, odds ratio, RR relative risk.
