# Supplementary material for: Fish Intake and Risk of Liver Cancer: A Meta-Analysis
Source: PLoS One. 2015 Jan 23;10(1):e0096102. doi: 10.1371/journal.pone.0096102 (PMC4304705; doi:10.1371/journal.pone.0096102)
Supplement: S2 Table — (DOCX) [file pone.0096102.s003.docx]

**Table S2 .** Characteristics of published case-control and cohort studies on total fish intake and risk of liver cancer.

| Study | Country | Design | Number of cases/subjects | Outcome | Fish intake assessment | Case assessment | Comparison | OR (95%CI) | Variables |
| --- | --- | --- | --- | --- | --- | --- | --- | --- | --- |
| La Vecchia, 1988 | Italy | Hospital based case-control | 151/1202 | HCC incidence | interview | Histological or AFP confirmation | NA | 0.75 (0.48-1.16) | Age and sex. |
| Hirayama, 1989 | Japan | Prospective cohort) | 123/NA  (sub-cohort of patients with liver cirrhosis) | Liver cancer mortality | NA | NA | Daily vs. not daily | 0.97(0.69-1.35) | ­None. |
| Fernandez, 1999 | Italy | Hospital based case-control | 428/8418 | liver cancer incidence | Interview | NA | ≥2 vs.＜1 servings/week | 1.0 (0.7-1.3) | Age, sex, BMI, area of residence, education, smoking and alcohol intake. |
| Kurozawa, 2004 | Japan | Prospective cohort | 401/110688 | HCC mortality | Interview or  Self-reported FFQ | Death certificates | Daily vs. ≤1-2 servings/week | 0.85 (0.62-1.18) ^a^ | Age, sex and history of liver diseases. |
| Talamini, 2006 | Italy | Hospital based case-control | 185/597 | HCC incidence | interview | Histological or cytological Confirmation  (78.2%) | ＞6.5 vs. ＜3.5 servings/week | 1.12(0.52-2.4) | Age, sex, center, education, place of birth, drinking habits, maximal lifetime alcohol intake, hepatitis viruses, and total energy intake. |
| Kanazir, 2010 | Serbia | Hospital based case-control | 45/135 | HCC incidence | Interview | Histological  confirmation | Weekly vs. rarely | 0.3(0.1-0.7) | Age and sex. |
| Wang, 2011 | China | Population based case-control | 1116/13511 | Liver cancer mortality | Questionnaire completed by relatives of cases | Death registration | ＞4 servings/week  vs.  ≤3servings/month | 0.72 (0.49-1.08) | Age, sex, education, physical exercise, job types, smoking, and intakes of alcohol, meat, fruit and tea. |
| Daniel, 2011 | USA | Prospective cohort | 586/492186 | Liver cancer incidence | Self-reported FFQ | Cancer registries | 21.4 vs. 3.6 g/1000 kcal | 0.86 (0.68-1.13) | Age, sex, BMI, education, marital status, family history of cancer, race, smoking, physical activity, MHT in women, and intake of alcohol, fruit, vegetables, total energy, poultry and red meat. |
| Sawada, 2012 | Japan | Prospective cohort | 398/90296 | HCC incidence | Self-reported FFQ | Cancer registries,  Death certificates or active patient notification | 160.6 vs. 35.0 g/d | 0.64(0.41-1.02) | Age, BMI, area, sex, smoking, history of diabetes, and intake of coffee, soy foods, vegetables, vegetable oil, protein, iron and alcohol. |
| Fedirko, 2013 | Ten  European  countries | Prospective cohort | 191/477206 | HCC incidence  and mortality | Self-reported FFQ | Histological  confirmation | ＞50.8 vs. 0-14.2 g/d | 0.63 (0.39-1.01) | Age, sex, BMI, study center, smoking, physical activity, diabetes status, lifetime alcohol intake pattern, and intakes of nonalcohol energy, coffee, alcohol, other types of meat, and dietary fiber. |

BMI, Body mass index; FFQ, food frequency questionnaires; HCC, hepatic carcinoma; MHT, menopausal hormone therapy; NA, not available.

^a^ The study reported relative risks by history of liver diseases, age and gender. The data reported here were the results combined with the fixed-effects model (*P* for heterogeneity=0.22 *I*^2^=26.8%)
